# A Comparative Retrospective Study of Immunotherapy RANO *Versus* Standard RANO Criteria in Glioblastoma Patients Receiving Immune Checkpoint Inhibitor Therapy

**DOI:** 10.3389/fonc.2021.679331

**Published:** 2021-06-25

**Authors:** Xin Chen, Mary Jane Lim-Fat, Lei Qin, Angie Li, Annie Bryant, Camden P. Bay, Lu Gao, Nityanand Miskin, Zaiyi Liu, J. Bryan Iorgulescu, Xiaoyin Xu, David A. Reardon, Geoffrey S. Young

**Affiliations:** ^1^ Department of Radiology, Brigham and Women’s Hospital, Boston, MA, United States; ^2^ Department of Radiology, Guangzhou First People’s Hospital, The Second Affiliated Hospital of South China University of Technology, Guangzhou, China; ^3^ Division of Neurology, Department of Medicine, Sunnybrook Health Sciences Center, University of Toronto, Toronto, ON, Canada; ^4^ Department of Imaging, Dana-Farber Cancer Institute, Boston, MA, United States; ^5^ Department of Radiology, Harvard Medical School, Boston, MA, United States; ^6^ Department of Family Medicine, University of California, Riverside School of Medicine, Riverside, CA, United States; ^7^ Department of Neurosurgery, Peking Union Medical College Hospital, Chinese Academy of Medical Sciences and Peking Union Medical College, Beijing, China; ^8^ Department of Radiology, Guangdong Provincial People’s Hospital, Guangdong Academy of Medical Sciences, Guangzhou, China; ^9^ Department of Pathology, Brigham and Women’s Hospital, Boston, MA, United States; ^10^ Center for Neuro-Oncology, Dana-Farber Cancer Institute, Boston, MA, United States; ^11^ Department of Medicine, Harvard Medical School, Boston, MA, United States

**Keywords:** glioblastoma, immunotherapy, magnetic resonance imaging, disease progression, pseudoprogression, response assessment in neuro-oncology criteria, immunotherapy response assessment for neuro-oncology

## Abstract

**Objectives:**

Real-time assessment of treatment response in glioblastoma (GBM) patients on immune checkpoint blockade (ICB) remains challenging because inflammatory effects of therapy may mimic progressive disease, and the temporal evolution of these inflammatory findings is poorly understood. We compare GBM patient response during ICB as assessed with the Immunotherapy Response Assessment in Neuro-Oncology (iRANO) and the standard Response Assessment in Neuro-Oncology (RANO) radiological criteria.

**Methods:**

49 GBM patients (seven newly diagnosed and 42 recurrent) treated with ICBs at a single institution were identified. Tumor burden was quantified on serial MR scans according to RANO criteria during ICB. Radiographic response assessment by iRANO and RANO were compared.

**Results:**

82% (40/49) of patients received anti–PD-1, 16% (8/49) received anti-PD-L1, and 2% (1/49) received anti-PD-1 and anti-CTLA4 treatment. Change in tumor burden and best overall response ranged from −100 to +557% (median: +48%). 12% (6/49) of patients were classified as concordant non-progressors by both RANO and iRANO (best response: one CR, one PR, and four SD). Another12% (6/49) had discordant assessments: 15% (6/41) of RANO grade progressive disease (PD) patients had iRANO grade of progressive disease unconfirmed (PDU). The final classification of these discordant patients was pseudoprogression (PsP) in three of six, PD in two of six, and PDU in one of six who went off study before the iRANO assessment of PDU. iRANO delayed diagnosis of PD by 42 and 93 days in the two PD patients. 76% (37/49) patients were classified as concordant PD by both RANO and iRANO. 12% (6/49) of all patients were classified as PsP, starting at a median of 12 weeks (range, 4–30 weeks) after ICB initiation.

**Conclusions:**

Standard RANO and iRANO have high concordance for assessing PD in patients within 6 months of ICB initiation. iRANO was beneficial in 6% (3/49) cases later proven to be PsP, but delayed confirmation of PD by <3 months in 4% (2/49). PsP occurred in 12% of patients, starting at up to 7 months after initiation of ICB. Further study to define the utility of modified RANO compared with iRANO in ICB GBM patients is needed.

## Introduction

Patients with glioblastoma (GBM), the most common primary malignant brain tumor in adults, face a poor prognosis, limited effective treatment options, and early clinical deterioration (1). Despite recent advances, current standard treatment with maximal surgical resection, and temozolomide chemoradiation yields a median overall survival (OS) of roughly 15 to 16 months in patients with newly diagnosed GBM (2, 3), and no salvage therapy has been proven to prolong OS. A number of immune-based strategies are being investigated in GBM, including immune checkpoint blockade (ICB), neoantigen vaccines, oncolytic viruses, and chimeric antigen receptor T cell therapy (4).

Over the past decade, immune checkpoint inhibitors targeting cytotoxic T lymphocyte-4 (CTLA-4; ipilimumab), programmed cell death protein 1 (PD-1; pembrolizumab, nivolumab, and cemiplimab), and programmed cell death ligand1 (PD-L1; durvalumab, atezolizumab, and avelumab) have obtained approval from the US Food and Drug Administration and become part of the standard of care for melanoma, non-small cell lung cancer, and other solid tumors based on impressive responses and prolonged OS in a minority of patients, some with very advanced disease (5–11). While ICB has produced promising responses in animal models of GBM (12), phase 3 human trials in both newly diagnosed GBM (CheckMate-498; press release, Bristol Myers Squibb, May 9, 2019, and Checkmate-548; press release, Bristol Myers Squibb, 12/23/2020) and recurrent GBM (CheckMate-143) (13) have failed to prolong OS. However, neoadjuvant use of both pembrolizumab (14) and nivolumab (15) has shown immunomodulatory effects, and neoadjuvant pembrolizumab appeared to have an OS benefit in a randomized multi-institutional cohort of 35 patients with recurrent GBM (14). Reports that the subpopulation of long-term responders to PD-1 ICB have tumors enriched in certain MAPK pathway alterations raises hope that ICB may prove effective in this or other targeted subpopulations, indicating the need research to discover biomarkers for identification of potential long-term responders before or early after ICB initiation (16). Biomarkers derived from clinical GBM patient MRI obtained soon after ICB initiation that are capable of differentiating patients likely to respond from patients unlikely to respond would be especially beneficial. In neuro-oncology, MRI assessment of GBM by the Response Assessment in Neuro-Oncology (RANO) criteria has been used in clinical trials since its inception in 2010 (17). RANO categorizes patients as having radiographic progressive disease (PD) when the tumor burden either (1) increases more than 25% when compared to MRI at baseline (defined as pre-therapy or best imaging response timepoint), or (2) when new lesions appear. However, in about 20% to 30% of patients treated with standard chemoradiation, treatment-related “pseudoprogression” (PsP) confounds this assessment by producing enhancement, edema, mass effect, and symptomatic worsening that subsequently resolve (18, 19). PsP is also observed following ICB, but the true rate and timing have not been clearly defined.

Because this early worsening of abnormality on MRI can lead to premature termination of therapy in potential responders and/or misinterpretation of clinical trial data, the revised immunotherapy RANO (iRANO) criteria were created in 2015 (20). iRANO differs from RANO in that per iRANO radiographic PD cannot be confirmed when the progressive imaging changes initially appear less than 6 months after starting immunotherapy. By iRANO such patients are classified as PD only after sustained worsening on MRI for three consecutive months. Although iRANO is designed to address ICB-related PsP, no published data exist that directly compare iRANO to RANO in ICB patients. Because that definition of response and progression is crucial when studying the effectiveness of novel immunotherapies, validation of iRANO is needed. We report direct comparison of RANO and iRANO assessments of ICB response and describe the patterns of response in a retrospective cohort of 49 GBM patients treated with ICB.

## Materials and Methods

### Patients

The institutional review board at our institution approved this retrospective analysis of patient records with a waiver of informed consent. Medical record data including MRI from 49 patients with GBM (seven newly diagnosed and 42 recurrent) treated in clinical trials of anti–PD-1 blockade with nivolumab (Bristol-Myers Squibb, Princeton, NJ, USA) or pembrolizumab (Merck, Kenilworth, NJ, USA), or anti-PD-L1 blockade with durvalumab (AstraZeneca Inc, NJ, USA) was retrieved. Patients with newly diagnosed GBM received standard treatment (maximal surgical resection, radiotherapy, and concomitant/adjuvant temozolomide) with biweekly nivolumab or pembrolizumab. One patient received dual checkpoint inhibition with ipilimumab and nivolumab alongside adjuvant temozolomide (ipilimumab once every 4 weeks for four courses and nivolumab every 2 weeks). Recurrent GBM patients received pembrolizumab every 3 weeks or nivolumab every 2 weeks, or durvalumab every 2 weeks, with or without bevacizumab. Patients were treated until disease progression, unacceptable toxicity, or voluntary withdrawal. MRI tumor assessment was performed every 8 weeks. As specified by RANO, a maximum of five target lesions were assessed per patient. All patients had baseline MRI prior to the initiation of ICB therapy and at least one follow-up MRI during ICB therapy.

### MRI Acquisition

MR images were acquired on 1.5 or 3T scanners (Siemens Erlangen, Germany and GE Healthcare, Waukesha, WI) using standard institutional protocols including axial FLAIR T2 and axial spin echo pre- and post-contrast T1-weighted images. The parameters in detail for MRI scans were as following: axial FLAIR T2WI, TR 8000-12ms; TE 550/81 to 135 ms; TI 2000 to 2650 ms; 3- to 5-mm slice thickness; gap 0 to 1 mm; matrix 256 to 384 × 244–288; axial spin echo pre- and post-contrast T1WI, TR 400 to 706ms; TE 2.5 to 17 ms; slice thickness 3 to 5 mm; gap 0 to 1 mm; matrix 256 to 384 × 192 to 244.

### Tumor Response Assessment According to iRANO and RANO

Imaging determinants of progression were assessed following published iRANO (20) and RANO (17) criteria. Clinical performance status was incorporated in our grading of PD. Data regarding steroid use were not available in all cases and so was not incorporated in the analysis. Per RANO, on baseline and all follow-up scans, the cross-product burden of abnormal enhancement was calculated as the sum of the products of the two largest perpendicular diameters of enhancing lesions on contrast-enhanced T1WI, and lesions with largest perpendicular diameter <10 mm and lesions visible on only one axial section were regarded as non-measurable excluded from analysis. Non-enhancing tumor and edema were not assessed.

Patients were classified as experiencing complete response (CR), partial response (PR), stable disease (SD), progressive disease (PD), and progressive disease unconfirmed (PDU) at each time point, according to RANO and iRANO. For patients meeting criteria for complete response (CR; i.e., complete disappearance of abnormal enhancement and no increase in non-enhancing abnormality on FLAIR T2WI) or partial response (PR; i.e., >50% decrease in cross-product enhancing tumor burden and no increase in non-enhancing abnormality on FLAIR T2WI), best overall response (BOR) was defined as the minimum enhancing lesion burden by the cross-product method during the trial. For patients meeting criteria for stable disease (SD; i.e., (<50% decrease and <25% increase in cross product tumor burden and no increase in non-enhancing abnormality on FLAIR T2WI) (17), the smallest cross-product was considered BOR only if abnormal enhancement remained stable on ICB 6 months from start of ICB.

iRANO criteria for SD, PR, and CR were the same as RANO, but PD criteria differ. Under iRANO, radiographic PD (i.e., >25% increase in burden of abnormal enhancement by cross product method or increase in FLAIR T2WI abnormality), identified within 6 months of starting ICB, was classified as unconfirmed progressive disease (PDU) until follow-up MRI 3 months after detection of PDU confirmed sustained progression on post-contrast and/or FLAIR T2WI (20). In addition, under RANO detection of a new measurable enhancing lesion constitutes PD by definition. Under iRANO, patients with a new enhancing measurable lesion were allowed to continue study therapy pending confirmation of progression on follow-up imaging. Finally, according to iRANO, patients who underwent resection or biopsy during the trial because of imaging evidence of worsening edema and enhancement were classified as PD if histopathologic assessment revealed a predominance of active viable tumor; alternatively, they were classified as SD if the specimen revealed predominantly necrosis, inflammation, and/or other treatment-related effects.

For RANO/iRANO, patients who required an increased dose of corticosteroids (usually dexamethasone) could not be defined as having achieved a response, while those who decreased dexamethasone prior to MRI and had progressive imaging changes should be deemed as non-evaluable. This component was, however, not included in our grading scheme.

For the purposes of this study, we defined pseudoprogression (PsP) as ≥25% increase in enhancing lesion cross-product compared with the smallest measurement since pretreatment baseline, when the enhancing lesion burden decreased on follow-up MRI or tissue pathology demonstrated predominantly necrosis, inflammation, and/or other treatment-related effect. PsP occurring <12 weeks after start of ICB was considered “early” PsP and PsP occurring >12 weeks after start of ICB was considered “late” PsP.

The authors were blinded to clinical assessment of progression or response. Percent agreement between iRANO and RANO for progression versus non-progression and 95% confidence intervals for agreement were calculated. Progression-free survival (PFS) was calculated from date of ICB initiation to date of progression or death by any cause, or if no progression was observed, from ICB initiation to the censored date. OS was calculated from start of initial therapy to death from any cause, or for patients still alive at the time of analysis, to the censored date. The last date of study follow-up was February 6, 2019.

### Statistical Analysis

Patients were classified into three groups based on difference between RANO and iRANO response assessment: group 1 (concordant non-progressors) includes patients with BOR of SD, PR, or CR by both RANO and iRANO; group 2 (discordant) includes patients with BOR of PD by RANO but PDU, SD, PR, or CR by iRANO; and group 3 (concordant progressors), patients with BOR of PD by both RANO and iRANO. Kaplan-Meier curves were plotted for the three groups, and pairwise log-rank tests were used to compare OS between the three groups. Individuals were right censored at the end of follow-up or if they voluntarily withdrew from the study; right censoring was considered non-informative. Testing was two-tailed, and a p-value < 0.05 was considered statistically significant. No correction was made to account for multiple hypothesis testing. Statistical analysis was conducted using R version 3.3.

## Results

### Patient Demographics and Treatment Characteristics

Median age at GBM diagnosis was 61 years (range, 26–81) (**Table 1**). 82% (40/49) of the patients received anti-PD-1 (nivolumab or pembrolizumab monotherapy), 16% (8/49) received anti–PD-L1 (durvalumab), and 4% (1/49) received dual ICB (nivolumab and ipilimumab). 82% (40/49) received ICB for recurrent GBM and 14% (7/49) as upfront therapy in newly diagnosed GBM. The Kaplan-Meier estimated median OS was 12.3 months (range, 3.1–43.8 months). Median time on therapy was 17.7 weeks (range, 2–208 weeks). Median follow-up time was 38 weeks (range, 13.4–187.7 weeks). One patient voluntarily withdrew from the study and was lost to follow-up. One patient was still receiving nivolumab therapy at the time of censoring.

**Table 1:** Patient demographics and clinical characteristics.

Characteristics	Variable (%)	All patients (n=49)	Group 1 Non-PD both criteria (n=6)	Group 2 PD per RANO, non-PD per iRANO (n=6)	Group 3 PD by both criteria (n=37)
Sex	Male	32 (65)	3 (50)	1 (17)	28 (76)
	Female	17 (35)	3 (50)	5 (83)	9 (24)
Age (years)	Median (range)	61 (26–81)	49 (41–68)	61 (57–69)	66 (26–81)
Type of tumor	New GBM	7 (14)	3 (50)	0 (0)	4 (11)
	Recurrent	42 (97)	3 (50)	6 (100)	33 (89)
Treatment	Nivolumab	10 (20)	3 (50)	3 (50)	4 (11)
	Pembrolizumab	30 (61)	3 (50)	2 (33)	25 (68)
	Durvalumab Nivolumab +	8 (16)	0 (0)	1 (17)	7 (19)
	Ipilimumab	1 (2)	0 (0)	0 (0)	1 (3)
IDH status	Wildtype	42 (86)	6 (100)	5 (83)	31 (84)
	Mutant	4 (8)	0 (0)	0 (0)	4 (11)
	Unknown	3 (6)	0 (0)	1 (17)	2 (5)
MGMT promoter	Methylated	15 (31)	3 (50)	1 (17)	12 (32)
	Unmethylated	16 (33)	3 (50)	1 (17)	11 (30)
	Partially methylated	7 (14)	0 (0)	1 (17)	6 (16)
	Unknown	11 (22)	0 (0)	3 (50)	8 (22)
Concurrent Bevacizumab Number of infusions	Yes	20 (41)	3 (50)	0 (0)	17 (46)
	No	29 (59)	3 (50)	6 (100)	20 (54)
	Median (range)	6 (2–76)	32 (8–76)	6.5 (2–18)	5.0 (2–32)

PD, progressive disease; RANO, Response Assessment in Neuro-Oncology; iRANO, Immunotherapy Response Assessment in Neuro-Oncology.

### Response Assessment by RANO and iRANO

**Figure 1:**
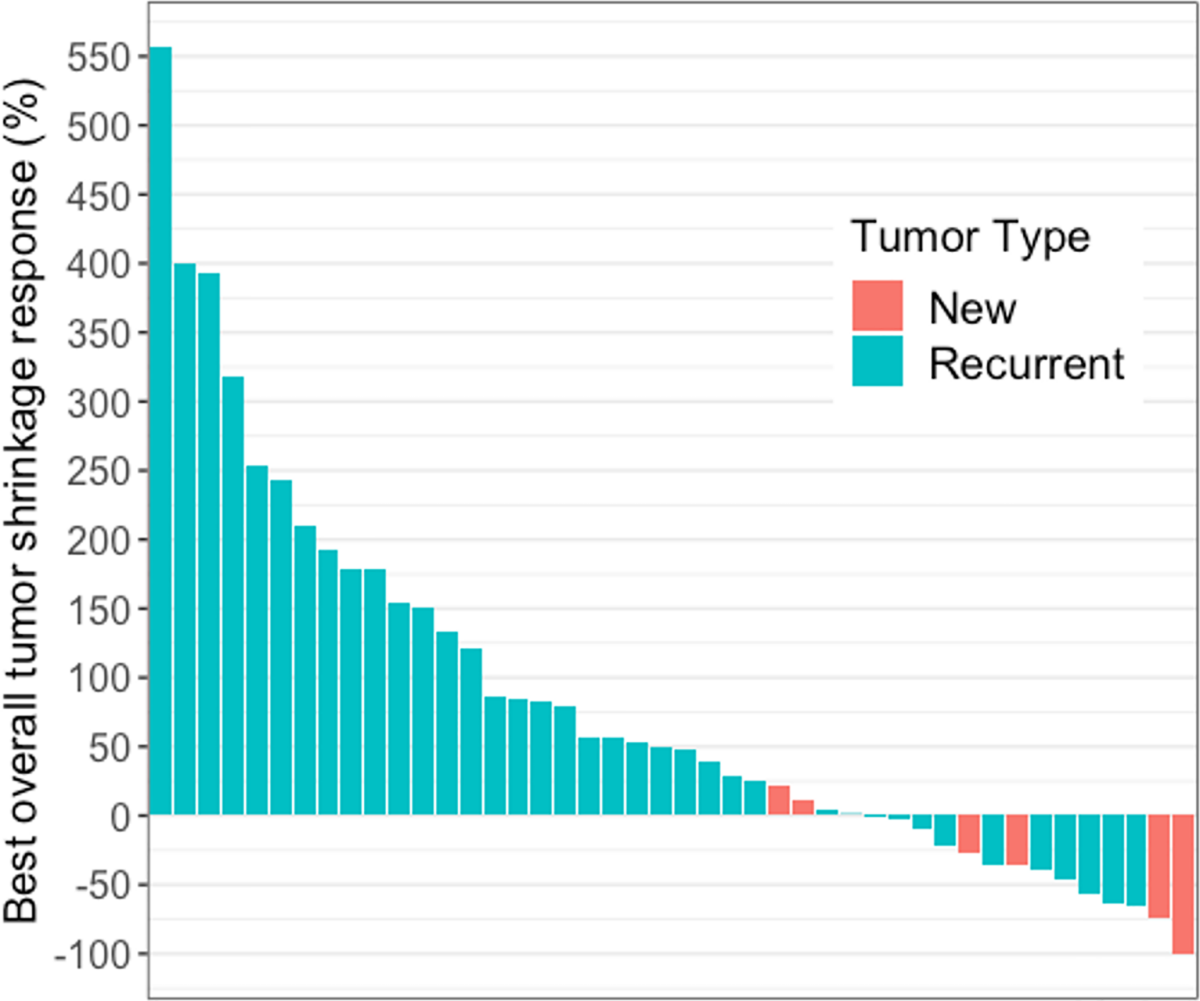
Waterfall plot showing best overall tumor shrinkage in the form of percentage change in enhancing lesion size compared to baseline in 44 of 49 patients with glioblastoma receiving immune checkpoint blockade. These 44 patients had measurable disease at baseline and the change in enhancing lesion burden at BOR, compared to baseline ranged from −100% to +557% (median: +48%).

Forty-four of 49 patients had at least one measurable enhancing lesion on pretreatment baseline MRI. In these 44 patients, the change in enhancing lesion burden at BOR, compared to baseline ranged from −100% to +557% (median: +48%) (**Figure 1**). The percentage agreement between iRANO and RANO for PD status was 88% (95% confidence interval [CI], 79% to 97%) (**Table 2**).

**Table 2:** Confusion matrix of PD status by iRANO and RANO (n = 49).

		RANO		
		PD	Non-PD	
iRANO	PD	37	0	37
	Non-PD	6	6	12
		43	6	49

PD, progressive disease; RANO, Response Assessment in Neuro-Oncology; iRANO, Immunotherapy Response Assessment in Neuro-Oncology.

12% (6/49) patients were stratified into group 1 (concordant non-progressors by both RANO and iRANO criteria); one CR, one PR, and four SD. Three of six patients in group 1 (one CR, one PR, one SD) received ICB upfront and the other three of six (three SD) received ICB for recurrence. 43% (3/7) of the upfront patients were in group 1, compared with 7% (3/42) of the recurrence patients.

12% (6/49) patients overall were stratified into group 2 (discrepant classification between RANO and iRANO), representing 15% (6/41) of the PD patients (**Tables 3A**, **B**). All six of these patients had PDU based on increasing burden of enhancing abnormality on MRI within 6 months ICB initiation. All six received ICB for recurrent GBM and none received concurrent bevacizumab with ICB. Half (3/6) were ultimately confirmed to have had PsP, two by pathology and one by later decrease in enhancement on MRI. PD was later confirmed in two of the other three patients by iRANO, at 42 and 93 days, respectively, after PD classification by RANO. The remaining one patient with PDU by iRANO was taken off trial after 3 months, and no follow-up scan obtained. 76% (37/49) of the patients were classified into group 3 (concordant PD by both RANO and iRANO).

**Table 3A:** Group 2 representing six patients with recurrent GBM with discordant PD date by RANO *vs* iRANO (PD by RANO and PDU by iRANO.

Patients	PsP	PD by RANO (trial day)	Date of confirmed PD or PsP (trial day)	Additional days on trial until confirmation of PD/PsP
4	Yes	29	92 (PsP: pathology)	63
7	No	70	112 (PD: pathology)	42
11	Yes	44	86 (PsP: imaging)	42
13	No	66	NA90 (no follow up scan)	NA
38	No	134	227 (PD: pathology)	93
46	Yes	68	150 (PsP: pathology)	82

Pathology denotes disease diagnosed by pathology specimen which revealed predominantly necrosis, inflammation, and/or other treatment-related effects.

PsP, pseudoprogression; NA, not available.

**Table 3B:** Group 2 representing six patients with recurrent GBM with discordant PD date by RANO *vs* iRANO (PD by RANO and PDU by iRANO.

Patient		Day 30	Day 60	Day 90	Day 120	Day 150	Day 180	Day 210	Day 240
4	RANO	PD							
	iRANO	PDU	PDU	PDU	PsP				
7	RANO	–	–	PD					
	iRANO	–	–	PDU	PD				
11	RANO	–	PD						
	iRANO	–	PDU	PsP					
13	RANO	–	–	PD					
	iRANO	–	–	PDU					
38	RANO	–	SD	SD	SD	PD			
	iRANO	–	SD	SD	SD	PDU	PDU	–	PD
46	RANO	–	–	PD					
	iRANO	–	–	PDU	PDU	PsP			

Scan dates were rounded to the nearest following month.

PD, progressive disease; PDU, PD unconfirmed; PsP, pseudoprogression; “-”, no scan.

### PsP During Immunotherapy

**Figure 2:**
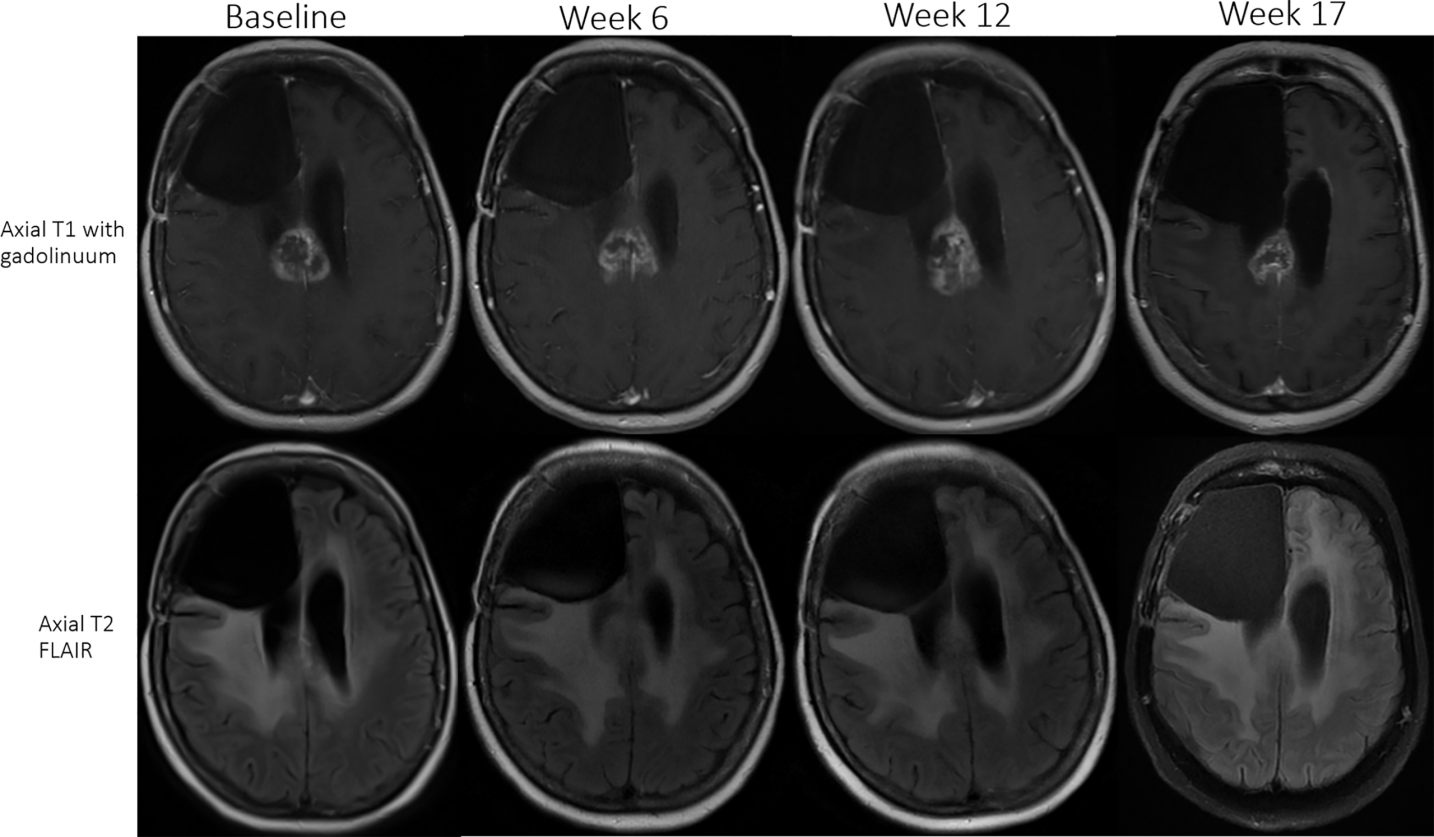
Patient 11 (received nivolumab and ipilimumab) had an initial 27% increase in enhancing lesion burden at first MRI (week 6), 13% decrease at the next follow-up MRI (week 12), and subsequently a 36% decrease (week 17) in size of the enhancing lesion. The patient remained stable on ICB for two additional months until treatment was discontinued due to declining performance status in the setting of a stable MRI scan.

12% (6/49) met the criteria for PsP (two receiving up-front ICB and four ICB for recurrence; **Table 4**). Median time from ICB initiation to initial detection of PsP was 12 weeks (range, 4–30 weeks). Three of six received nivolumab, one of six received nivolumab and ipilimumab, and two of six received pembrolizumab. MRI of patient 11 (nivolumab and ipilimumab) demonstrated a 27% increase in enhancing lesion burden at first MRI (week 6), 13% decrease on next follow-up MRI (week 12), additional 36% decrease on the next MRI (week 17), after which, the burden of enhancing abnormality remained stable for two additional months until ICB was discontinued due to declining performance status (**Figure 2**). MRI of patient 6 (nivolumab) revealed 31% increase in enhancing lesion burden at 30 weeks from the start of ICB, and no change at 38 weeks after which histopathology confirmed predominant treatment effect. MRI of the other four PsP patients (pembrolizumab or nivolumab) demonstrated progressive increase in enhancing lesion burden leading to re-resection at 4 to 30 weeks with pathology showing extensive treatment-related effects.

**Table 4:** Pseudoprogression characteristics in 6/49 glioblastoma patients.

Patient	Type	Treatment	Time to PsP (weeks)	OS／time spent on trial (weeks)	Confirmed
4	recurrent	N	4	23/12	Pathology
6	new	N	30	70/66	Pathology
9	new	N	23	104/59	Pathology
11	recurrent	N+I	6	73/27	Follow-up
43	recurrent	P	6	35/19	Pathology
46	recurrent	P	10	55/41	Pathology

Pathology denotes disease diagnosed by pathology specimen which revealed predominantly necrosis, inflammation, and/or other treatment-related effects. Follow-up denotes confirmed by stable disease of follow-up imaging.

N, nivolumab; I, ipilimumab; P, pembrolizumab; PsP, pseudoprogression; OS, overall survival.

### New Lesions During Immunotherapy

16% (8/49) of the patients (one up-front ACB and seven ICB for recurrence) developed one to three new lesions per patient during ICB (**Table 5**). In four of eight, none of the enhancing lesions were large enough to be measurable.

**Table 5:** Characteristics of new lesions during immunotherapy.

Patient	type	treatment	Time (weeks)	# of lesion(s)	iRANO	Confirmed results	OS (weeks)
9	new	N	55	1 non-measurable	PD	PD	104
13	R	D	9	1 measurable	PDU	PDU	23
16	R	D	24	1 measurable	PDU	PDU	61
18	R	D	11	3 non-measurable	PD	PD	24
21	R	P+B	25	1 non-measurable	SD	PD	77
24	R	P+B	10	1 non-measurable	SD	PD	27
28	R	P+B	14	2 non- and 1 measurable	PD	PD	16
41	R	P+B	2	1 measurable	PDU	PD	13

Time was measured from initial i—ICB to appearance of new lesion.

new, newly diagnosed GBM; R, recurrent GBM; N, nivolumab; D, durvalumab; P, pembrolizumab; B, bevacizumab; SD, stable disease; PD, progressive disease; PDU, PD unconfirmed; OS, overall survival.

### Survival Outcomes

**Figure 3:**
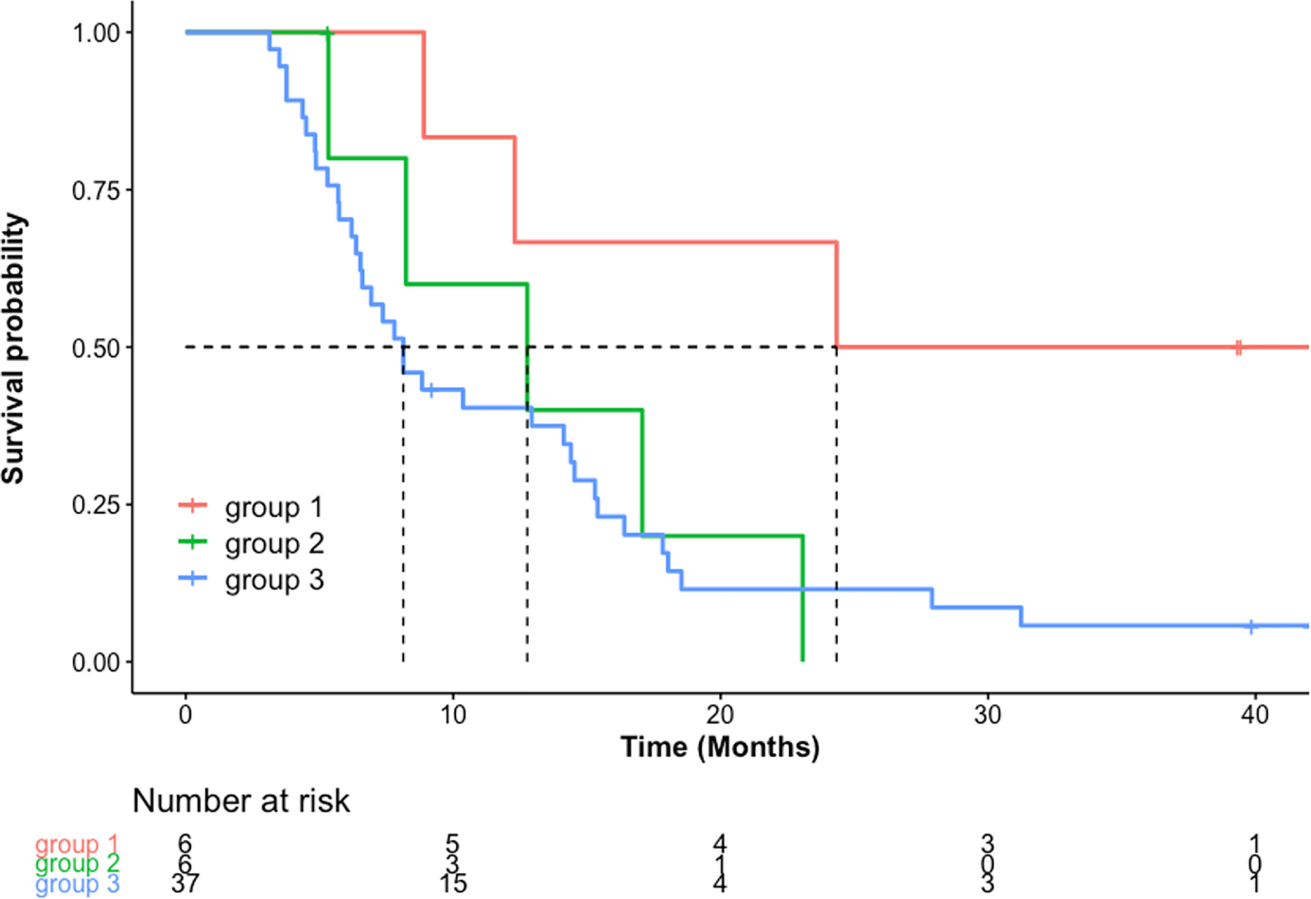
OS in months [Kaplan-Meier estimated median and 95% confidence interval (CI), months] was significantly longer in group 1 [24.3 (12.3-not estimable)] compared to group 2 [12.8 (8.2 to not estimable)] (p=0.05) and group 3 [8.1 (6.5–14.5) (p=0.01)]. There was no significant difference in median OS between group 2 and group 3 (p=0.7).

OS was significantly longer in the concordant non-progression group 1 (median, 24.3 months; 95% CI, 12.3 to not estimable) compared with discordant group 2 (median, 12.8 months; 95% CI, 8.2 to not estimable; p < 0.05) and concordant progression group 3 (median, 8.1; 95% CI, 6.5–14.5; p = 0.01) (**Figure 3**). There was no difference in OS between group 2 and group 3 (p = 0.7). Median PFS as assessed by RANO was 2.7 months (1.9–3.8 months) and median PFS by iRANO was 3.7 months (2.9–5.6 months).

The median OS in the newly diagnosed GBM patients treated with up-front ICB was 24.3 months (95% CI, 15.4 to not estimable) compared with 8.2 months (95% CI, 6.9–14.1 months) in patients treated with ICB for recurrent GBM. Using the RANO criteria, median PFS was 8.9 months (95% CI, 6.5 to NA) in newly diagnosed GBM compared to 1.9 months (95% CI, 1.9 to 2.8 months) in recurrent GBM. Using the iRANO criteria, the median PFS was 8.9 months (95% CI, 6.5 to NA) in the up-front ICB group compared with 3.3 months (95% CI, 2.8–4.7; p = 0.003) in ICB for recurrence. Patients experiencing PsP did not differ in OS (median, 14.6 months; 95% CI, 8.1 to NA) compared with the other patients (median, 8.9 months; 95% CI, 6.9–15.3; p = 1.0).

## Discussion

In order to simulate typical prospective clinical application of MRI response criteria, our retrospective analysis of MRI in 49 GBM patients receiving ICB compared response assessment by iRANO criteria with the standard RANO criteria. The wide range of observed changes in enhancing lesion burden (−100% to +556.6%) (**Figure 1**) was consistent with known heterogeneous patient response to PD-1 or PD-L1 ICB among GBM and other cancer patients (21–23). We found a high concordance between RANO and iRANO. Concordant non-progression was determined in 12% (6/49) of the patients, and progression in 76% (37/49) by both criteria, for a substantial percentage agreement of 88%. Non-progressors by either RANO or iRANO had better survival than patients who were classified as PD by both criteria and better survival than patients with discordant grading by RANO and iRANO. This indicates that a subset of GBM patients have sustained response to ICB, which confers a survival advantage. In our cohort, the median PFS was 2.7 months using standard RANO and 3.7 months using iRANO, but a subgroup of long-term responders also had PFS of 24.4 months (patient 5), 26.1 months (patient 45), 35.6 months (patient 1), and 37.5 months (patient 10). We did not design our study to account for expected differences in survival between newly diagnosed and recurrent GBM and only a small subset of our cohort received up-front ICB for newly diagnosed GBM. Nevertheless, two interesting patterns suggest the possibility that up-front ICB patients may derive more benefit from ICB compared with patients receiving ICB for recurrence. We did not design our study to account for expected differences in survival between newly diagnosed and recurrent GBM and only a small subset of our cohort received up-front ICB for newly diagnosed GBM. Nevertheless, the significantly longer PFS and OS and higher rate of concordant non-progression in the newly diagnosed group (50% in group 1 compared to 0% in group 2 and 12% in group 3) deserves additional study in larger up-front cohorts to assess whether up-front ICB may be more effective than ICB at recurrence.”

MRI assessment by iRANO differs from standard RANO principally in that PD is only confirmed by iRANO when (1) the increasing enhanced tumor burden on MRI first appears at or later than 6 months after immunotherapy initiation, or (2) enhancing lesion burden continues to increase on follow-up MRI > 3 months after initial detection. This is based on studies in other solid tumors demonstrating that increasing enhancing lesion burden stabilized or improved within 3 months in patients who were ultimately found to derive benefit from ICB (24, 25).

In two of 49 patients subsequently confirmed to have PD by pathology, MRI within the first 6 months demonstrated increasing enhancement, which was classified as PD by RANO but PDU by iRANO. The differences in the criteria and/or lack of follow-up imaging 3 months following the initial MRI with increasing enhancement led to delay in confirmation of progression and extension of time on trial by 42 and 93 days, respectively, after initial PD determination by RANO. It is unclear whether earlier identification and withdrawal from the clinical trial would have provided any clinical benefit, because no effective salvage therapy exists for GBM recurrence. On the other hand, the use of iRANO allowed maintenance of ICB in 3 of 49 patients who were later found to have PsP, extending their progression-free time on trial for 42 to 82 days. These patients may have benefited from use of iRANO. However, it is worth noting that there was no difference in overall survival in patients in group 2 or group 3, and that in this context, using iRANO, while more precise, may not reflect any difference in underlying tumor biology. If in the future, more effective therapies are developed for patients with recurrent disease, the significance of differences between iRANO and RANO may require re-analysis because the relative delay in assignment of PD by iRANO could have the unintended effect of delaying transition to effective salvage therapy.

The incidence of PsP was 12% in our cohort, which is lower than previously reported rates of PsP in GBM after temozolomide chemoradiation (18, 19). This may in part be due to the fact that most patients in our cohort had recurrent GBM (42/49, 86%), a group in which true progression is more common than PsP. Indeed, 28.5% (2/7) of the patients with newly diagnosed GBM and 9.5% (4/42) of the patients with recurrent GBM in our cohort experienced PsP. PsP was noted initially between 4 and 10 weeks after ICB initiation in patients with the ICB for recurrence group, and 23 to 30 weeks in the up-front ICB group. This is consistent with a published analysis of ICB in a multi-institutional cohort of 152 GBM patients reporting that immunotherapy-related inflammation is less common than progressive disease within 6 months of ICB initiation (26). These data suggest that inflammatory PsP in immunotherapy may present differently in newly diagnosed compared with recurrent ICB; the optimal ideal timeframe and monitoring strategy may differ between these groups. The OS in patients with PsP was not significantly different from those without PsP. This finding was consistent with that of the previously cited data presented at ASCO 2020, which showed no difference in post-progression OS between patients with and without prior immune-related inflammation (26). These data raise the possibility that the potential clinical benefit of ICB may be partially masked by the morbidity of ICB-induced PsP, and emphasizes the importance of ongoing efforts to modulate the inflammatory effects of immunotherapy.

Of the 16% (8/49) of the patients who developed new lesions in our cohort, two of eight initially met iRANO criteria for SD. Six of eight were eventually confirmed to have PD on follow-up MRI, but one of two patients with PDU by iRANO survived longer than 1 year after PDU, in spite of discontinuation of immunotherapy based on RANO criteria at the time of new lesion appearance. It is unclear if this patient or several other patients in which ICB was discontinued based on RANO criteria would have had benefited from additional ICB if iRANO criteria had been used instead, but this suggests the hypothesis that iRANO may make an important difference to a small number of patients. The role of iRANO remains to be further elucidated in the context of the anticipated rise of more effective immunotherapeutic strategies both in the upfront and recurrent setting.

Limitations of our study inherent to its retrospective design include the heterogeneous population of newly diagnosed and recurrent GBM patients, different combinations of prior tumor-directed therapies, and lack of sufficient follow-up scans to confirm progression in some patients. A larger population of patients will be needed to identify imaging or other prognostic factors predictive of ICB response. In addition, while both standard RANO and iRANO include information on corticosteroid use, we did not include this. This weakness leads to possible under-estimation of the theoretical accuracy of the criteria but accurately reproduces clinical MRI practice in which reliable steroid dosing data are rarely available at time of image interpretation. Further, availability of steroid dose information would not have changed MRI assessment of PD by either RANO or iRANO, and most clinical trials do not allow for continuation of ICB after significant increase in corticosteroid dose because of evidence that steroid use may abolish the benefit of ICB. Lastly, we did not include the modified RANO (mRANO) criteria in this study. mRANO was developed to take into account PsP and adopts a strategy of intermediate rigor between RANO and iRANO by requiring confirmation of PD on a subsequent scan at the next scheduled time point, after which, if there is further increase in tumor size, PD is backdated to the preliminary PD scan (27). In addition, if stable disease or partial response is seen on the subsequent follow up scan, mRANO grades the response as PsP. In short mRANO represents a more recent rational adaptation of RANO that may improve performance, but the ideal timing of the follow-up confirmatory scan after preliminary PD remains to be defined in future studies.

In conclusion, MRI assessment of anti-PD-1 and anti-PD-L1 ICB of GBM in our retrospective cohort suggests a high concordance of RANO and iRANO. iRANO may provide important benefit by identifying PsP, seen in 12% of our cohort. Use of iRANO delayed identification of PD in two of 49 cases prolonging time on trial for these patients compared with RANO. The utility of these criteria may need to be assessed if immunotherapies are developed, which are more effective in a subgroup or all GBM patients, or if imaging or genomic markers can be identified that predict higher probability of PsP, early progression, and/or substantial ICB benefit in a subgroup of patients. This retrospective study cannot determine whether these patients would have benefited clinically from use of RANO or conversely if several other patients discontinued from ICB based on RANO would have benefited from use of iRANO leading to continuation of ICB. Recent mRANO criteria may be better adapted for patients on ICB, allowing for identification of PsP while also allowing for early confirmation of PD, although the timeframe for follow up scans remains unclear. Imaging markers predictive of GBM patient subgroups with high probability of PsP, early progression, and/or substantial benefit from ICB deserve further study in larger cohorts with combined imaging, clinical, and genomic datasets.

## Data Availability Statement

The raw data supporting the conclusions of this article will be made available by the authors, without undue reservation.

## Ethics Statement

The studies involving human participants were reviewed and approved by Institutional Review Board of Brigham and Women’s Hospital. The patients/participants provided their written informed consent to participate in this study. Written informed consent was obtained from the individual(s) for the publication of any potentially identifiable images or data included in this article.

## Author Contributions

XC, ML-F, LQ, GY, and DR participated in the conception and study design, data acquisition and review of the images and measurements, statistical analysis and interpretation of data, drafting and revising the manuscript. AL, AB, LG, and NM participated in the data acquisition and review of the images and measurements. CB participated in the statistical analysis and revising the manuscript. ZL, XX and JI participated in the interpretation data and revising the manuscript. All authors contributed to the article and approved the submitted version.

## Funding

This study was supported with US National Institutes of Health (NIH R01LM012434) [XX-Salary, GY-Salary], the National Key R&D Program of China (2017YFC1309100) [ZL-Salary], the National Science Fund for Distinguished Young Scholars (81925023) [ZL-Salary], the National Natural Scientific Foundation of China (81601469 [XC-Salary], 82072090 [XC-Salary], 81771912 [ZL-Salary]), Guangzhou Science and Technology Project of Health (20191A011002) [XC-Salary], China Scholarship Council funding (201808440033 [XC-Salary], 201706210406 [LG-Salary]), the National Cancer Institute (K12CA090354) [BI-Salary] and the Conquer Cancer Foundation/Sontag Foundation [BI-Salary].

## Conflict of Interest

The authors declare that the research was conducted in the absence of any commercial or financial relationships that could be construed as a potential conflict of interest.

## Abbreviations

GBM, glioblastoma; OS, overall survival; ICB, immune checkpoint blockade; CTLA-4, cytotoxic T-lymphocyte-associated protein 4; PD-1, programmed death receptor 1; PD-L1, programmed death-ligand 1; NSCLC, non-small cell lung cancer; RANO, response assessment in neuro-oncology; PD, progressive disease; iRANO, immunotherapy RANO; CR, complete response; PR, partial response; BOR, best overall response; SD, stable disease; PDU, progressive disease unconfirmed; PsP, pseudoprogression; PFS, progression free survival.
